# Hypercalcemia as the Initial Presentation of Acute T-cell Leukemia/Lymphoma

**DOI:** 10.7759/cureus.23705

**Published:** 2022-03-31

**Authors:** Vignesh Ramachandran

**Affiliations:** 1 Department of Dermatology, New York University, New York, USA; 2 Department of Internal Medicine, Texas Health Presbyterian Hospital, Dallas, USA

**Keywords:** general internal medicine, inpatient care, pathophysiology, clinical pathology, adult t-cell leukemia/lymphoma, hypercalcemia

## Abstract

Adult T-cell leukemia/lymphoma (ATLL) is a T-cell malignancy that generally presents with widespread involvement of lymph nodes, peripheral blood, and/or skin. It is an uncommon malignancy linked to the human T-lymphotropic virus 1 (HTLV-1). Herein, we present a case of ATLL that was diagnosed after a patient presented to our hospital with nonspecific symptoms of fatigue and weakness and was subsequently found to have hypercalcemia secondary to his blood malignancy. We engage in a discussion of the etiology, epidemiology, and management of patients with this rare malignancy as well as the mechanisms that result in hypercalcemia.

## Introduction

Hypercalcemia is a common reason for admission to the inpatient setting and is classically defined by a calcium level above 10.5 mg/dL, 40% of which is ionized form [1]. Hypercalcemia can lead to numerous complications and sequelae, including acute kidney injury, arrhythmia, acute pancreatitis, constipation, neurological impairments, and even coma [2-4]. Oftentimes, patients may not endorse symptoms specific to a particular etiology and a broad workup may be necessary. Within the broad range of differential diagnoses, hematologic malignancies should be included. A retrospective chart review of intensive care unit admissions for severe hypercalcemia (defined as calcium > 12 mg/dL) at an academic institution revealed that 44% of cases were related to hematologic malignancy [5]. The diagnosis can be difficult to make without a high index of suspicion, which may be triggered by means of presenting symptoms, history, and workup. An uncommon cause of hypercalcemia is adult T-cell leukemia/lymphoma (ATLL), which is associated with human T-lymphotropic virus 1 (HTLV-1) infection [6]. Herein, we illustrate a case of ATLL diagnosed in a patient presenting with nonspecific complaints and hypercalcemia in addition to engaging in a discussion of the etiology, epidemiology, and management of patients with this rare malignancy as well as the mechanisms that result in hypercalcemia.

## Case presentation

A 52-year-old Nigerian-born male without significant past medical history presented to our hospital with chief complaints of weakness and fatigue. He states that his symptoms started approximately one month prior and were brought to his attention by his peers, who noticed he appeared more labored at work. He also reported lower leg bone pain and weight loss, which was partially intentional but not to the extent to account for his nearly 40-pound weight loss over the last few months. He denies fevers, chills, night sweats, changes in appetite, rash or other symptoms. He did not have a primary care physician and had not seen a provider in decades. Family history was notable for two siblings with unknown malignancies, one of whom was diagnosed during childhood. He denied smoking history, alcohol use, or intravenous drug use.

**Figure 1:**
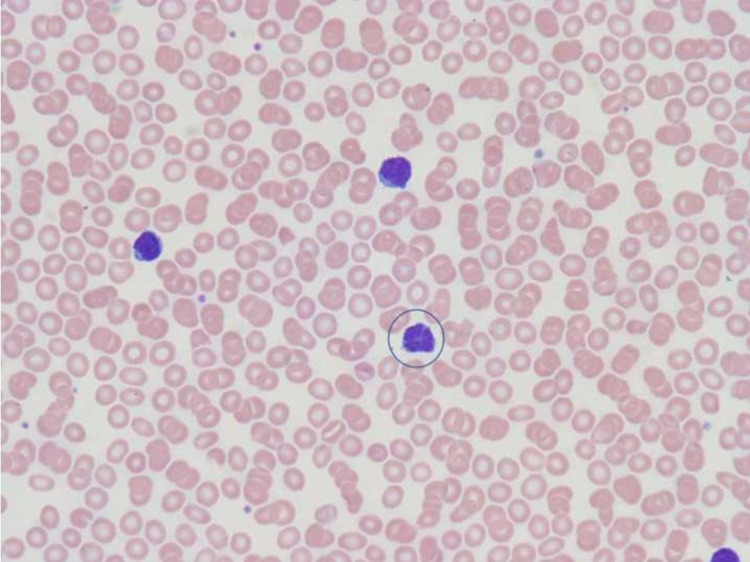
Peripheral blood smear (600X magnification) demonstrating the characteristic “flower cell” of adult T-cell leukemia/lymphoma, which has a convoluted multilobate nucleus.

Upon admission, he was afebrile, borderline tachycardic to 99 beats/minute (regular rhythm), hypertensive (172/99 mm Hg), and breathing comfortably on room air. Physical examination was notable for a slightly palpable spleen and mild tenderness to palpation of the lower legs. Initial laboratory workup was initiated with a complete blood count and complete metabolic panel. White blood cell count was elevated to 15.91 cells/L [reference: 4.5 to 11.0 × 10^9^/L] with a lymphocytic predominance (10.82%) while creatinine was elevated to 2.04 mg/dL in association with an elevated calcium of 15.6 mg/dL [reference: 8.6-10.3 mg/dL]. The remaining laboratory values of the complete blood count and complete metabolic panel were within normal limits. A hypercalcemia workup was initiated and a peripheral blood smear was also sent. From this workup, the notable result was that parathyroid hormone-related peptide (PTH-rp) was elevated to 10.9 [normal <2.5 pmol/L]. Serum protein electrophoresis was normal. Computerized tomography studies of the chest, abdomen and pelvis revealed mild perihepatic lymphadenopathy. The peripheral blood smear revealed white blood cells that were increased in number and demonstrated a predominant population of atypical (95%) small to medium mature lymphoid cells with clumped chromatin, irregular/flower-like nuclei, inconspicuous nucleoli and scant eosinophilic cytoplasm (Figure 1). A bone marrow biopsy was subsequently performed revealing 70% cellularity and 10% nodular lymphoid aggregates composed of atypical lymphoid cells similar to those seen in the peripheral blood smear. Immunohistochemistry revealed positivity for CD2, CD3, CD4, CD5 (weak positive), and CD30 (focal positivity). HTLV I and II testing was sent out and HTLV I was found to be positive by Western blot assessment.

Overall, these findings were consistent with those of adult T-cell leukemia/lymphoma (ATLL). In light of the hypercalcemia, splenomegaly, neoplastic lymphocytosis, and mild abdominal lymphadenopathy, the acute phase of ATLL was favored.

During the workup, the patient was managed via aggressive intravenous fluid resuscitation and one dose of 60 mg pamidronate (intravenous), which led to a marked decrease in calcium to normal range levels over the course of three days of admission.

## Discussion

HTLV-1 most often causes asymptomatic infection and, as a result, leads to silent transmission via sexual intercourse, breastfeeding and blood transfusions. There are no vaccines against this virus and screening of blood banks (although required by the Food and Drug Administration in the United States) and during prenatal care is not universally performed worldwide. HTLV-1 is most prevalent in Africa, Caribbeans, South America, Central America, and Asia. Two major illnesses it can cause are ATLL and HTLV-associated myelopathy, which is also known as tropical spastic paraparesis [7]. 

ATLL is a T-cell malignancy that generally presents with widespread involvement of lymph nodes, peripheral blood, and/or skin. The course can be indolent or can be aggressive. It is linked to infection with the HTLV-1 virus [8]. It is an uncommon neoplasm with incidence varying by prevalence of HTLV-1 infection. In the United States, the incidence is 0.05 cases per 100,000 persons with slight male predominance and average age of diagnosis in the sixth decade [9,10]. The proposed pathophysiology implicates T-regulatory cells which become infected by the HTLV-1 virus. Malignant transformation occurs with the activation of oncogenes (tax gene product [TAX] and hemoglobin subunit zeta [HBZ] are implicated), inactivation of tumor suppressor genes, DNA damage and replication of viral microRNA. Over an incubation period of 30-50 years, it is thought that the sequelae of these deleterious effects manifest as ATLL [11].

Hypercalcemia is uncommonly the presenting sign in patients with ATLL, however it may be quite severe in these individuals. It is thought to be paraneoplastic secondary to chemokines secreted by malignant cells, parathyroid hormone-related peptide, tumor necrosis factor beta or interleukin-1, and increased receptor activator of nuclear factor kappa-Β ligand (RANKL) [12]. It is important to keep ATLL in the differential for a patient with hypercalcemia especially in the setting of a peculiar complete blood count with lymphocytic predominance as clinical symptoms may be nonspecific or the course may be indolent. Additionally, social history, particularly travel history, or place of birth may aid in diagnosis as some regions have a higher prevalence of HTLV-1 as was illustrated in our patient’s history.

Amidst our global pandemic, such insidious diseases may go undetected, especially in patients not connected to our healthcare systems. In this vein, telemedicine can be used to ensure patients get regular lab work and have access to primary care services where these conditions can be caught initially.

## Conclusions

Adult T-cell leukemia/lymphoma is an uncommonly encountered pathology in the inpatient setting, especially as a new diagnosis. It is especially an intriguing differential to consider when a patient presents with hypercalcemia. While hypercalcemia typically can be seen as a heralding sign of solid tumors due to paraneoplastic syndromes, this case represents the importance of maintaining similar suspicion of hypercalcemia for hematologic malignancies, particularly ATLL in the setting of lymphocytosis. The simple but effective use of a peripheral blood smear in this scenario could crucially benefit providers with differentiation of neoplastic lymphocytosis leading to early recognition and diagnosis of this uncommon pathology.
